# Deciphering the Acetabular Labrum's Cellular Atlas: MDK Inhibition as a Novel Therapeutic Method for Developmental Dysplasia of the Hip

**DOI:** 10.1002/advs.202505803

**Published:** 2025-07-02

**Authors:** Runze Yang, Huiling Liu, Minghao Ge, Dingyuan Zhang, Tianhao Xu, Lei Zhang, Yanlin Zhu, Shun Li, Jian Li, Xin Ma, Weili Fu

**Affiliations:** ^1^ Sports Medicine Center Department of Orthopedic Surgery/Orthopedic Research Institute West China Hospital Sichuan University Chengdu Sichuan 610064 China; ^2^ School of Life Science and Technology University of Electronic Science and Technology of China Chengdu Sichuan 610054 China; ^3^ Tianfu Jiangxi Laboratory Chengdu Sichuan 641419 China; ^4^ GenomCan Inc. Chengdu Sichuan 610040 China

**Keywords:** acetabular labrum, developmental dysplasia of the Hip, midkine, single‐cell RNA sequencing, spatial transcriptome sequencing

## Abstract

DDH is far more than just a contributing factor to hip osteoarthritis; it represents a formidable challenge to the health of infants and young children. While historical research has largely focused on the osseous abnormalities associated with DDH, current surgical interventions, though prevalent, often carry significant and unavoidable side effects. To break through these limitations, the study delved into the acetabular labrum, a crucial intra‐articular structure that presents abnormalities in DDH patients. Through single‐cell combined spatial transcriptomic analysis of the acetabular labrum under diverse conditions, a comprehensive examination of the changes of DDH tissue changes at the single‐cell level is pioneered, conclusively linking aberrant fibrocartilage stem/progenitor cell proliferation to disease progression. The subsequent in vivo and in vitro experiments unequivocally identify the MK signaling pathway as a pivotal target for DDH treatment. Localized administration of its specific inhibitor not only substantially alleviates early structural abnormalities in DDH but also lays a robust and promising foundation for the innovation and clinical translation of new therapeutic strategies for DDH. In summary, the findings not only deepen the understanding of DDH pathogenesis but also pave the way for innovative therapeutic strategies that prioritize both efficacy and safety.

## Graphical abstract

This study explores developmental dysplasia of the hip (DDH) by analyzing acetabular labrum abnormalities using single‐cell and spatial transcriptomics. Aberrant fibrocartilage stem cell proliferation is linked to DDH progression. Targeting the MK signaling pathway with a specific inhibitor effectively alleviates early DDH abnormalities, offering promising therapeutic potential and advancing understanding of DDH pathogenesis.

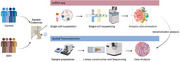

## 1 Introduction

Developmental Dysplasia of the Hip (DDH) is a pivotal contributor to childhood disability, characterized by distinct developmental anomalies within the hip joint structure. These anomalies encompass irregular acetabulum morphology, insufficient acetabulum coverage, and atypical femoral head‐acetabulum positioning.^[^ ^1^ ^]^ Such aberrant development predisposes individuals to hip instability, elevates the risk of injury, and instigates progressive degenerative changes. Ultimately, this progression culminates in hip osteoarthritis, severely compromising hip joint functionality and resulting in a marked incidence of joint disability.^[^ ^2^ ^]^ Alarmingly, DDH accounts for a staggering 29% of primary conditions necessitating hip replacement surgery in the middle‐aged and elderly cohort, imposing substantial socioeconomic strain.^[^ ^3^ ^]^ Traditionally, DDH has been conceptualized as predominantly stemming from skeletal structural abnormalities, emphasizing physical correction as the linchpin of effective treatment.^[^ ^4^ ^]^ Present therapeutic strategies predominantly hinge on early‐stage orthotic correction and stabilization, with surgical intervention reserved for advanced manifestations.^[^ ^5^ ^]^ However, the sustained efficacy of orthotic interventions remains nebulous, with patient's adherence to rigorous protocols proving challenging. Moreover, surgical avenues, while effective, are marred by invasiveness and substantial financial implications, amplifying the emotional and economic toll on patients and families alike.^[^ ^6^ ^]^ In light of these complexities, there exists a pressing imperative for pioneering treatment modalities tailored to DDH. Currently, molecular therapy and targeted treatment, renowned for their minimal invasiveness and favorable side‐effect profiles, have witnessed burgeoning exploration across diverse medical domains, including cancer, osteoporosis, and neurodegenerative disorders.^[^ ^7^ ^]^ Regrettably, their applicability in the DDH therapeutic landscape remains conspicuously uncharted, highlighting an urgent and promising frontier for innovative therapeutic approaches in this intricate clinical context.

The advancement of innovative treatment approaches in DDH hinges on a deeper understanding of its underlying pathogenic mechanisms. The multifaceted etiology of DDH involves a myriad of factors, including genetic predisposition, fetal malposition, and the maternal uterine milieu.^[^ ^8^ ^]^ Moreover, factors like congenital hip joint instability, fetal positioning in the uterine cavity, and placental location can also influence the onset of DDH.^[^ ^9^ ^]^ Recent studies have increasingly delved into non‐skeletal components of the hip joint, particularly focusing on the acetabular labrum.^[^ ^10^, ^11^ ^]^ This triangular fibrocartilaginous structure attaches to the acetabulum's edge,^[^ ^12^ ^]^ encircling it much like a horseshoe, deepening it and thereby expanding the femoral head's coverage and bolstering joint stability.^[^ ^13^ ^]^ Remarkably, up to 90% of symptomatic DDH cases manifest labral pathology, characterized by thickening, enlargement, and tearing of the labral tissue.^[^ ^11^ ^]^ Research indicates that DDH patients bear 4–5 times the load on the acetabular labrum compared to healthy individuals, making it more prone to degeneration and thus accelerating the onset of hip osteoarthritis.^[^ ^14^ ^]^ Literature suggests that preserving the acetabular labrum can mitigate and delay the progression of hip osteoarthritis in DDH surgical patients.^[^ ^15^ ^]^ Yet, a gap remains as no prior studies have examined DDH's etiology from the perspective of the acetabular labral angle. Recognizing the acetabular labrum's pivotal role in hip biomechanics and health, we posit that a comprehensive exploration of its microenvironmental changes in DDH, coupled with a deeply dive into the mechanism underpinning these alterations, will provide crucial insights for the pioneering novel DDH therapies.

The cellular composition of the acetabular labrum is heterogeneous, predominantly comprising fibrochondrocytes, alongside stem cells, vascular endothelial cells, macrophages, lymphocytes, and more. Constructing a comprehensive cellular landscape of the labrum is essential for deciphering its alterations in DDH. Studies indicate findings such as chondrocyte apoptosis, intra‐articular ossification, mast cell and macrophage infiltration, and vascular endothelial hyperplasia in the acetabular labrum of patients with severe hip osteoarthritis.^[^ ^16^ ^]^ With the continuous advancements in orthopedic tissue engineering, researchers have gained deeper insights into orthopedic‐related stem cells. Among these, fibrocartilage stem/progenitor cells (FSPC), a type of multipotent stem cell or progenitor cell capable of differentiating into chondrocytes, are thought to play a crucial role in the pathogenesis and treatment of injuries and degeneration of structures such as the meniscus and intervertebral discs.^[^ ^17^, ^18^ ^]^ However, the role of FSPC in DDH remains unclear. Recent whole‐exome sequencing studies on DDH have highlighted the critical role of low‐density lipoprotein receptor‐related protein 1 (LRP1) in its pathogenesis. *LRP1* defects impede autophagy and disrupt chondrocyte differentiation, precipitating DDH onset.^[^ ^19^ ^]^ Furthermore, mutations in *CXC3R1*, *IL‐6*, *TGF‐β1*, and *PAPPA2* have been shown to be closely linked to the DDH pathogenesis.^[^ ^20^ ^]^ Nonetheless, the spectrum of involved cell types, the primary cells driving the disease, and potential therapeutic targets for DDH remain elusive. Single‐cell RNA sequencing (scRNA‐seq) and spatial transcriptome sequencing are robust technologies for probing transcriptomic variations across individual cells.^[^ ^21^ ^]^ Despite their promise, these cutting‐edge techniques have yet to be applied in DDH research, offering unparalleled resolution in identifying distinct cell populations and shedding light on both physiological and pathological processes. Consequently, the integration of these advanced technologies heralds a new frontier in addressing the complexities of DDH.

In this study, we collected control acetabular labrum samples from patients with femoral head osteonecrosis and DDH acetabular labrum samples from DDH patients. We then performed scRNA‐seq and spatial transcriptome sequencing analysis to investigate DDH pathogenesis from the acetabular labrum perspective. For the first time, we've assembled a single‐cell transcriptome atlas encompassing all major human acetabular labrum subtypes, identifying chondrocyte subset trajectories during labrum degeneration. In addition to the analytical results derived from sequencing data, we have also deliberately developed an interactive website designed to store and allow readers to browse our processed data (https://bitbybit.shinyapps.io/shinysc/). To delve deeper into DDH therapeutic targets, we isolated and analyzed FSPC, revealing hyperplasia in the DDH patient acetabulum labrum and linking DDH progression to the MK signaling pathway via FSPC interaction. Through in vivo and in vitro experiments with the specific MDK inhibitor iMDK, we found that localized iMDK therapy can ameliorate or even reverse early structural DDH issues.

In summary, our research indicates that targeting the MK signaling pathway in early interventions could present a groundbreaking approach to treating DDH. Additionally, our findings offer pivotal guidance for the clinical translation of cutting‐edge therapeutic strategies for DDH, potentially unlocking new avenues for addressing fibrocartilage‐related conditions such as intervertebral disc and meniscus degeneration. Expanding upon these insights, our study sets a cornerstone for the progression of precision medicine in managing intricate musculoskeletal disorders, heralding more precise and impactful therapeutic interventions in the coming years.

## 2 Results

### 2.1 The Cellular Landscape of the Human Acetabular Labrum in Different States

**Figure 1:**
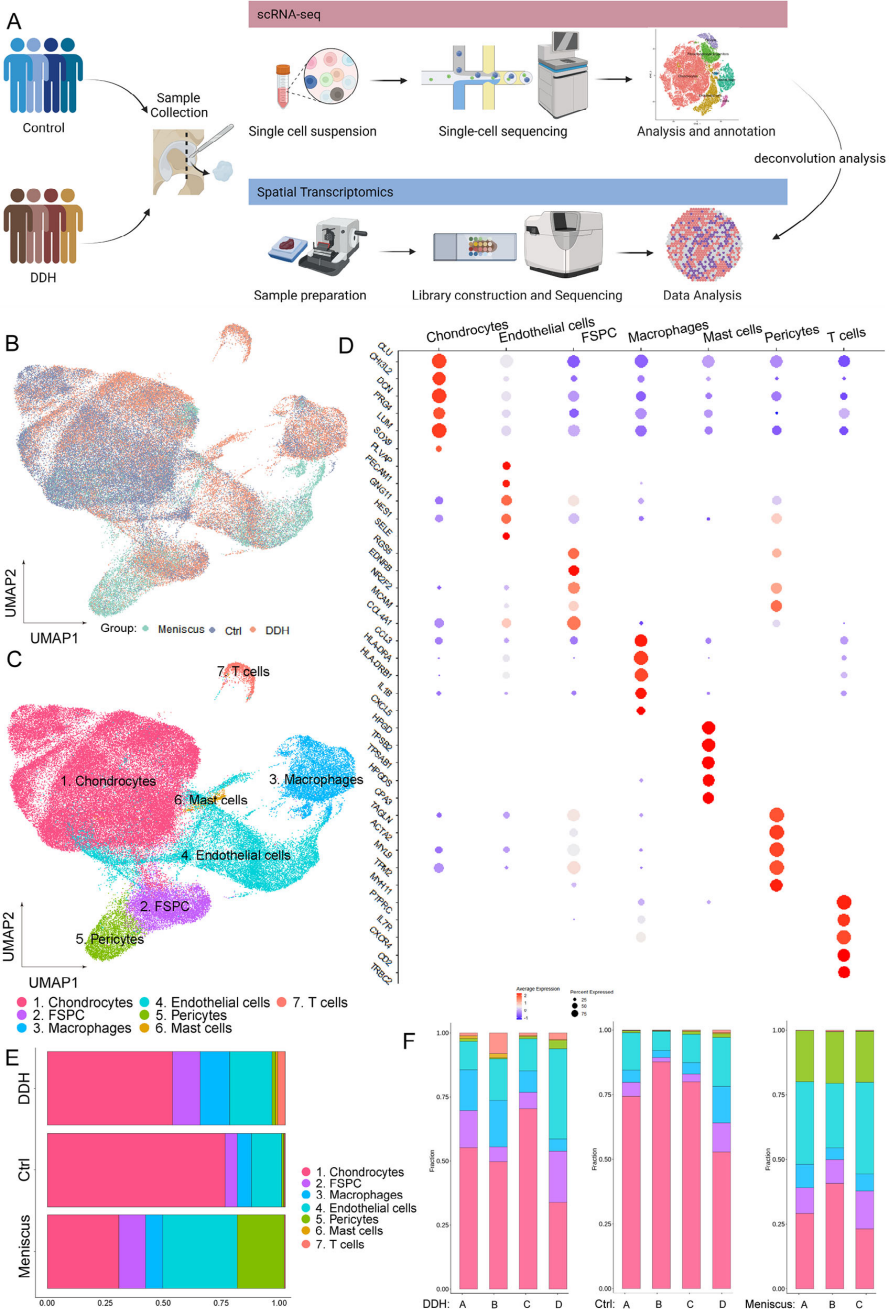
Single‐cell RNA‐seq reveals major cell classes in human acetabular labrum. A) The overall workflow of our research programme. B) UMAP visualization of the donor origins in meniscus, control, and DDH samples. C) UMAP visualization of all cell clusters in collected specimens. D) Dot plot of selected marker genes in each cell cluster. E) The percentages of the identified cell clusters in meniscus and control and DDH acetabular labral samples. F) The proportion of cell subsets at each sample level.

To delve deeper into the underlying pathogenesis of DDH, we collected four acetabular labrum samples from patients with osteonecrosis of the femoral head to serve as the control group, alongside four samples from patients with DDH as the diseased group, followed by performing both scRNA‐seq and spatial transcriptome sequencing (**Figure** **1**A). Clinical assessment utilizing magnetic resonance imaging (MRI) of the hip revealed a notable enlargement of the acetabular labrum in the DDH group compared to the control group (Figure S1, Supporting Information). Acknowledging the challenge of procuring entirely normal human acetabular labrum specimens, we supplemented our dataset with sequencing data from three healthy human outer menisci obtained from a prior study, considering the structural similarity between the outer meniscus and the acetabular labrum.^[^ ^22^ ^]^ Despite the potential concern that including meniscal tissue might expand the study's complexity, we wish to emphasize that the central focus of our analysis remains on delineating the developmental mechanisms underlying DDH. The meniscus served only as an auxiliary reference point to characterize the non‐degenerative state of fibrocartilaginous tissue. After meticulous quality control procedures, including the removal of low‐quality cells and inferred doublets, we obtained transcriptomes from a total of 88 989 cells (meniscus:13 781, control group:39 814, DDH group:35 394, Figure S2A,B, Supporting Information).

Initially, Uniform Manifold Approximation and Projection (UMAP) was employed to identify major cell compartments in all samples. As shown in Figure 1B, cells from different sample sources exhibited distinct distributions on the UMAP plot, particularly in chondrocytes, which differed markedly between the control and DDH groups. Meanwhile, we observed seven cell compartments, including chondrocytes, macrophages, mast cells, pericytes, T cells, FSPC, and endothelial cells (Figure 1C). These cell types were determined based on established lineage‐specific marker genes (Figure 1D). We classified cell clusters as chondrocytes if they highly expressed cartilage‐related genes such as *CLU*, *CHI3L2*, *LUM*, *SOX9*, and *DCN*, and those with high expression levels of *PLVAP*, *PECAM1*, and *SELE* as endothelial cells. Clusters showing high expression levels of stem cell‐related markers *RGS5*, *NR2F2*, *MCAM*, and *EDNRB* were classified as FSPC. Pericytes were identified by their expression of *TAGLN*, *ACTA2*, *MYL9*, and *TPM2*. Immune cells were recognized using macrophage markers *HLA‐DRA*, *HLA‐DRB1*, mast cell markers *TPSB2*, *TPSAB1*, and T cell markers *PTPRC*, *CD2*, *TRBC2*. Subsequently, we analyzed the proportions of all cell clusters in these three groups. Stacked bar plots in Figure 1E,F revealed that, in general, chondrocytes were the main cell type in the meniscus and acetabular labrum, with the meniscus group exhibiting the highest endothelial cell ratio. Compared with the control group, the DDH group had a higher number of FSPC, endothelial cells, macrophages, and pericytes.

### 2.2 Characterization of Chondrocytes and FSPC Subpopulations

**Figure 2:**
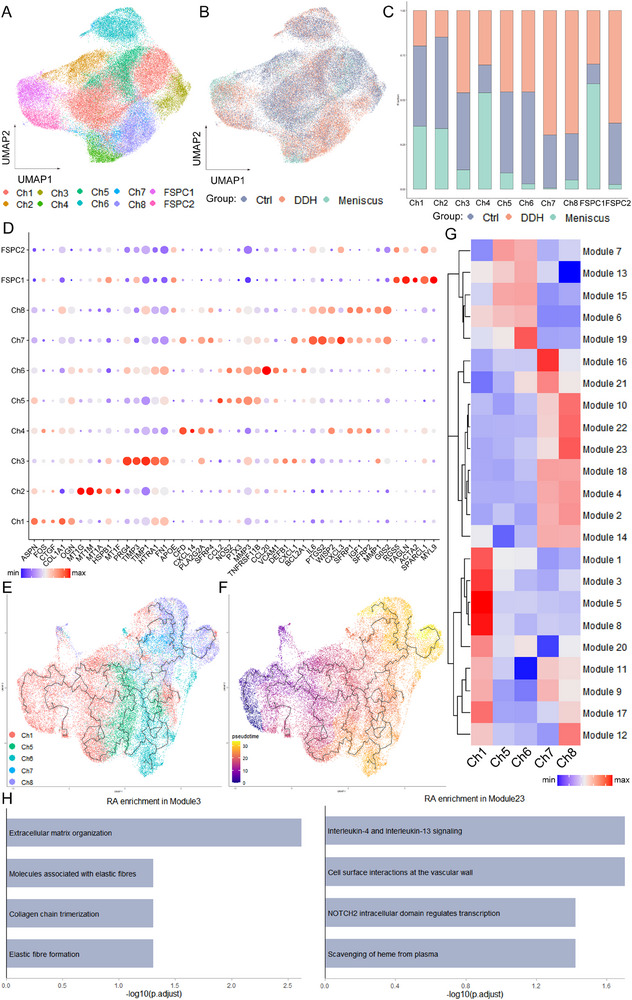
Characterization of chondrocyte subclusters and evolution trajectory during acetabular labral degeneration progression. A) UMAP visualization of the subpopulations of chondrocytes and FSPC. B) UMAP visualization of the distribution of chondrocyte subclusters in meniscus, control, and DDH samples. C) The proportions of 10 identified subpopulations in meniscus, control, and DDH groups. D) A bubble plot reveals the normalized expression of DEGs for each subcluster defined in. E,F) UMAP visualization of Ch1, Ch5, Ch6, Ch7, and Ch8. Developmental pseudotime for cells present along the trajectory inferred by Monocle 3, with the starting point of Ch1. G) Heatmap showing the scaled mean expression of modules of coregulated genes grouped by Louvain community analysis across the subclusters. H) Enrichment analysis results of Modules 3 and 23.

Given the pivotal roles of chondrocytes, the most abundant cell population in the acetabular labrum, and FSPC in the pathogenesis of DDH, we conducted a detailed analysis of these two cell clusters across all samples. As illustrated in **Figure** **2**A,B, we identified eight subpopulations of chondrocytes and two subpopulations of FSPC, each exhibiting distinct distribution on the UMAP plot across different sample groups. The proportion of chondrocyte subclusters varied significantly among the different groups, as depicted in Figure 2C (Figure S3A, Supporting Information). Specifically, the control group exhibited a higher proportion of Ch1, Ch2, and Ch6, whereas the DDH group showed a high proportion of Ch7, Ch8, and FSPC2. However, in the meniscus group, Ch4 and FSPC1 had a relatively high proportion.

To delineate the characteristics of chondrocyte subpopulations, we conducted differentially gene expressing analysis (DEGs) within these eight clusters (Figure 2D). Combined with the results of the proportion analysis, we observed distinct functional profiles for each chondrocyte subpopulation. Specifically, Ch1 represents a group of chondrocytes associated with extracellular matrixes (ECM) synthesis, functioning normally as they highly expressed genes related to ECM components such as *ASPN*, *CTGF*, *PRELP*, and *COL1A1*.^[^ ^23^ ^]^ Ch2, expressing metallothionein family genes such as *MT1G*, *MT1M*, *MT1A*, and *MT1E*, constitutes a population of tissue homeostasis‐associated chondrocytes with defense and antioxidant functions.^[^ ^24^ ^]^ Ch3 comprises lubrication‐associated chondrocytes, demonstrating high expression of *PRG4*, *TIMP3*, and *TIMP1*.^[^ ^25^ ^]^ Ch4 is characterized as a group of chondrocytes related to complement activation, exhibiting a high expression level of complement‐related genes such as *CFD*, *CXCL12*, *C3*, and *C7*. Ch5 and CH6 express genes associated with inflammation and chemotaxis, including *CCL2*, *NOS2*, *PTX3*, *TNFRSF11B*, and *CCL20*, indicative of their role in chondrocytes chemotaxis and inflammation.^[^ ^26^ ^]^ As for the remaining chondrocytes clusters, we empirically defined Ch7 (expressing *IL6*, *PTGS2*, and *WISP2*) as pre‐hypertrophic chondrocytes, and Ch8 (expressing *SFRP1*, *IGF1*, *SFRP2*, and *SFRP4*) was identified as hypertrophic chondrocytes.^[^ ^17^ ^]^

### 2.3 The Trajectory of Chondrocyte Transformation in the Process of Tissue Degeneration

Given that the acetabular labrum specimens we collected are from middle‐aged and elderly patients, their tissues inevitably undergo varying degrees of degeneration. Moreover, based on the proportion of degeneration‐related chondrocyte subsets (Ch7, Ch8) between the DDH group and the control group, it can be concluded that the degree of acetabular labrum degeneration in the DDH group is more severe. To gain insight into the cellular progression of subclusters of chondrocytes during acetabular labrum degeneration, we conducted cell trajectory and RNA velocity analysis, revealing an important pathway in the process of degeneration. Five chondrocyte subpopulations, including Ch1, Ch5, Ch6, Ch7, and Ch8, were included in the analysis using Monocle 3 and RNA velocity to reconstruct disease trajectories. We designated Ch1 as the starting point of the trajectory due to its representation of resident chondrocytes expressing a high level of ECM component genes, reflecting the normal function of the acetabular labrum. We then assigned pseudotime values to cells along the inferred developmental axis (Figure 2E,F; Figure S3B, Supporting Information). The results illustrated a pseudotime route from Ch1 to Ch5 and Ch6, and Ch7 and Ch8 located at the end of the trajectory. The results of RNA velocity analysis also illustrated that there existed a degeneration trajectory along the Ch1‐Ch5‐Ch6‐Ch7‐Ch8 path (Figure S3G, Supporting Information).

To further investigate gene expression dynamics along the trajectory, Louvain community analysis was employed to group genes into 23 modules across different cell clusters. The results in Figure 2G depict the aggregated expression of each module. Notably, we observed a gradual decrease in the expression of genes in module 3 along the trajectory, which enriched results related to Extracellular matrix organization and Collagen chain trimerization. In contrast, the expression of genes related to interleukin‐4 and interleukin‐13 signaling, associated with osteogenesis, showed a gradual increase along the trajectory (Figure 2H). Based on these pseudotime analysis results, we inferred a process of osteogenesis during the degeneration of the acetabular labrum. The expression changes of representative genes in module 3 and module 23 further supported this conclusion (Figure S3C,D, Supporting Information).

### 2.4 Identification of Subclusters of Human FSPC

**Figure 3:**
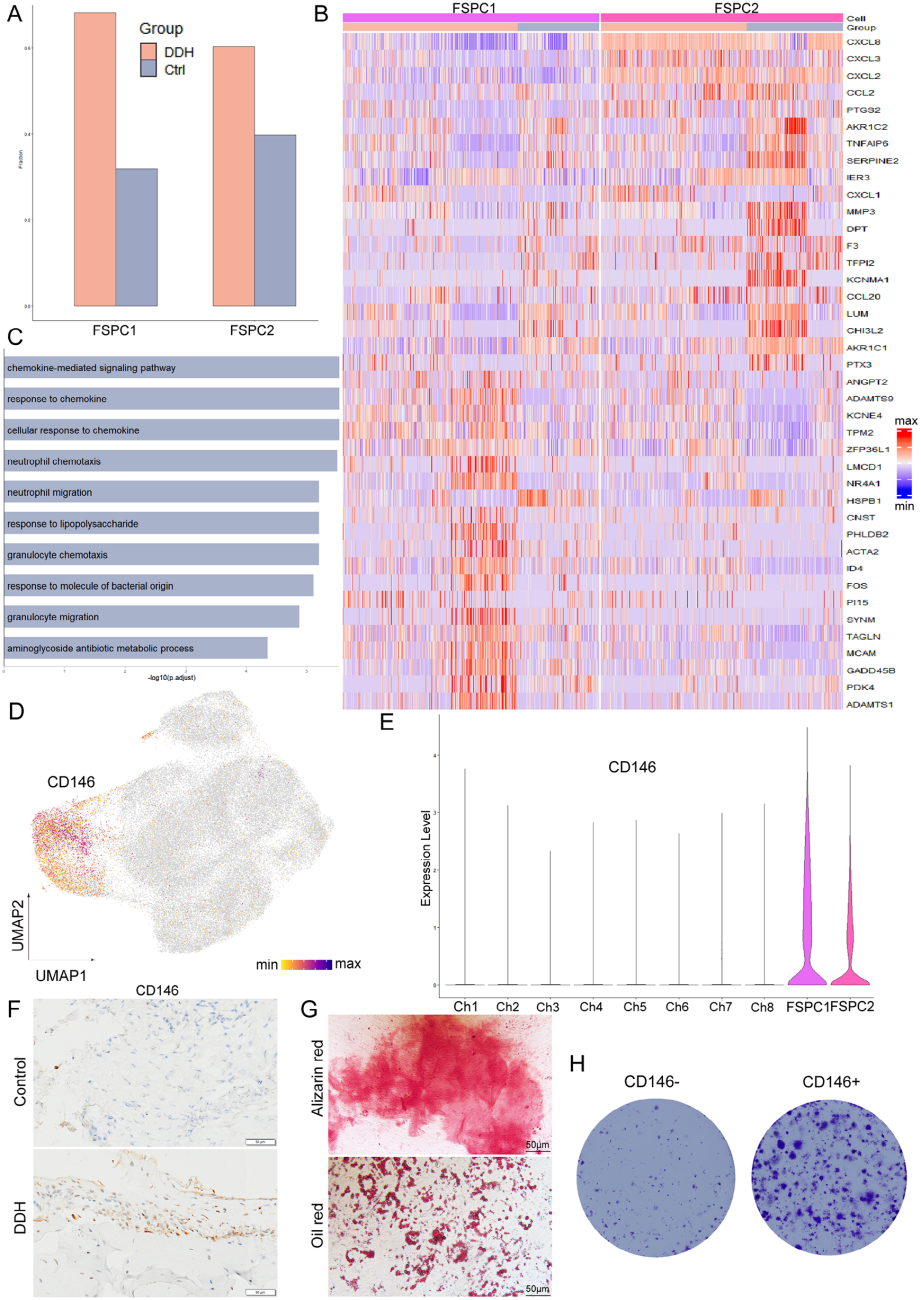
Characterization and identification of human fibrocartilage stem/progenitor cells. A) The proportion of two FSPC subclusters in the control and DDH groups. B) Heatmap showing the Degs between FSPC1 and FSPC2. C) GO enrichment analysis of Degs highly expressed by FSPC2. D,E) Dot plots showing the CD146 expression on UMAP map and Vin plot. F) Immunohistochemical staining for CD146 in control and DDH samples. G) Alizarin red staining and oil red staining for CD146+ acetabular labrum cells induced to osteogenic differentiation or adipogenic differentiation in order to prove the potential for multi‐lineage differentiation. H) Colony‐forming analysis of CD146+ and CD146− human acetabular labrum cells.

To further comprehend the characteristics of identified FSPCs and determine their role in DDH, we conducted an in‐depth analysis of these two subclusters. Analyzing the proportion of FSCP subsets in the control and DDH groups revealed that both FSPC subsets in the DDH group were significantly higher than those in the control group (**Figure** **3**A). DEGs analysis between the two subpopulations indicated that FSPC2 exhibited a higher expression level of genes involved in chemotaxis, such as *CXCL8*, *CXCL3*, *CCL2*, and *CXCL2* (Figure 3B). The Gene Ontology (GO) and GSEA enrichment results of DEGs illustrated that the increased expression of genes in FSPC2 was enriched for the GO terms such as “chemokine‐mediated signaling pathway,” “response to chemokine,” and “neutrophil migration,” while the decreased genes in FSPC2 were enriched for terms like “muscle contraction” and “muscle system process” (Figure 3C; Figure S3E,F, Supporting Information). This suggests that FSPC2 population cells may have a function in immune cell chemotaxis, while FSPC1 population cells may be related to contraction.

FSPC highly expressed the mesenchymal stem cell marker MCAM (CD146) (Figure 3D,E). Immunohistochemical staining of CD146 on acetabular labrum tissue in the two groups corroborated these findings, showing significantly higher expression of CD146 in the DDH group compared to the control group (Figure 3F). Semi‐quantitative analysis of the immunohistochemical results using ImageJ software also supported this conclusion (Figure S4A, Supporting Information). Employing CD146 fluorescent staining and flow cytometry, we isolated CD146+ primary human acetabular labrum cells. Subsequently, alizarin red and oil red O staining demonstrated that CD146+ cells possessed the ability to differentiate into various cell lineages, including osteoblasts and adipocytes (Figure 3G). Furthermore, we examined the clonogenicity of CD146+ cells. Seeding 1000 CD146+ cells and 1000 CD146‐ in 6‐well plates and culturing them for 7 days revealed a significantly higher number of colonies in the CD146+ group compared to the CD146‐ group (Figure 3H). These results demonstrated that the cells sorted by labeling CD146 exhibited the ability for stem/progenitor cell differentiation and proliferation.

### 2.5 Putative Signaling Network Regulating the Progression of DDH

**Figure 4:**
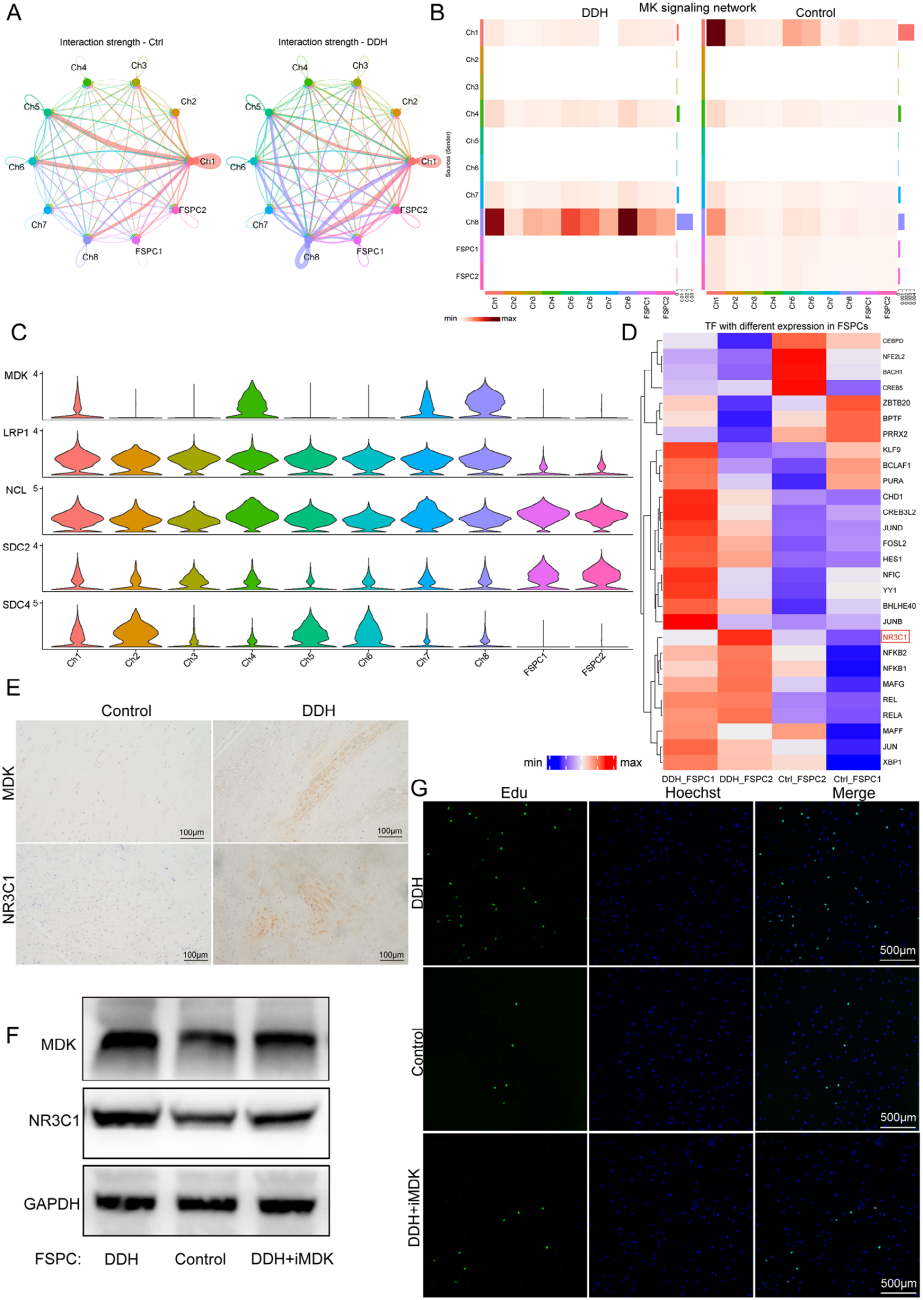
Cell–cell crosstalk in control and DDH groups. A) Circle plots showing the strength of interaction among the identified subclusters of chondrocytes and FSPC in control and DDH groups. B) Heatmap depicting the inferred MK signaling network in the two groups. C) Violin plots showing ligand‐receptor interactions related to MK signaling network among the subpopulations of chondrocytes and FSPC. D) SCENIC analysis of two subclusters of FSPC in DDH and control groups. E) Immunohistochemical staining for MDK and NR3C1 in control and DDH samples. F) Western blot showing the expression level of MDK and NR3C1 in FSPC of different states. G) Edu staining of FSPC in different groups.

To elucidate the explicit interaction among subclusters of chondrocytes and FSPCs in the microenvironment of the human acetabular labrum, shedding light on the underlying mechanisms of DDH pathogenesis, we employed Cellchat analysis. The circle plots illustrated that compared with the control group, the intensity of cell interaction was higher in the DDH group, with enhanced cell‐cell cross‐talks mainly concentrated between Ch8, FSPC1, FSPC2, and other clusters. Clinical observation has noted the obvious thickness and hypertrophy of the acetabular labrum in DDH patients compared to the healthy tissue. Additionally, these abnormal changes in the acetabular labrum are closely related to DDH progression. The results of Cellchat analysis also revealed specific differential interaction pathways between the DDH group and the control group. We observed significant activation of the MK signaling network, closely related to cell proliferation, in the DDH group, mainly present between Ch8 and other cells (Figure S3H, Supporting Information; **Figure** **4**B). Furthermore, FSPCs with proliferative capacity are crucial receptor cells in the MK signaling network (Figure 4C). Therefore, we inferred that the up‐regulated expression of *MDK*, the ligand of the MK signaling pathway, in the DDH microenvironment promotes the proliferation of FSPC, leading to the hyperplasia and hypertrophy of the acetabular labrum, thereby promoting DDH progression.

Subsequently, single‐cell Regulatory Network Inference and Clustering (SCENIC) analysis was performed to deeply decipher the difference in FSPCs between the control group and the DDH group. As is shown in Figure 4D, the DDH group exhibited a higher level of *NR3C1* compared to the control group. *NR3C1* has been reported to positively regulate cell proliferation, and the activation of *MDK* promotes the high expression of *NR3C1*. We further examined the expression level of MDK and NR3C1 in the two sample groups, which were consistent with the scRNA‐seq results (Figure 4E; Figure S4B,C, Supporting Information). After that, we examined the NR3C1 and MDK expression levels and cell proliferation in primary FSPCs derived from different groups and treated with iMDK, an inhibitor of MDK. The results illustrated that compared with the DDH group, the control group exhibited decreased expression levels of NR3C1 and MDK and decreased cell proliferation. However, compared with the DDH group, the expression level of NR3C1 and MDK, as well as cell proliferation, decreased in FSPCs treated with iMDK (Figure 4F,G; Figure S4D–F, Supporting Information).

### 2.6 Spatial Transcriptome Sequencing of the Human Acetabular Labrum

**Figure 5:**
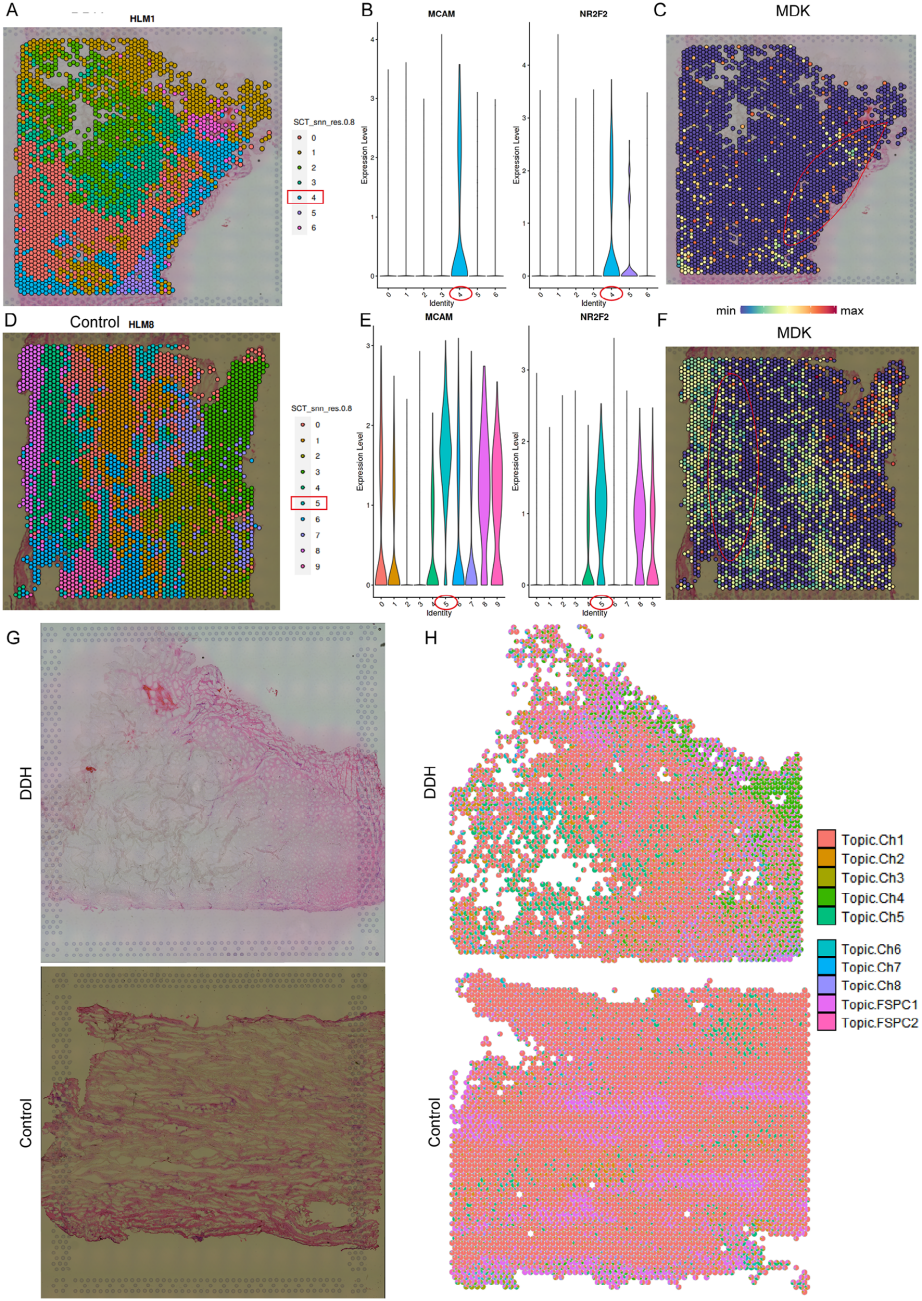
Spatial transcriptome sequencing in human acetabular labrum. A) Distribution of the 7 cell subsets identified in spatial transcriptome sequencing of the DDH group. B) Violin plots showing the expression of MCAM and NR2F2 in the identified 7 cell clusters in the DDH group. C) Spatial heatmap showing the expression of MDK in the DDH sample. The red wireframe area is the area of inferred stem/progenitor cell distribution. D) Distribution of the 10 cell subsets identified in spatial transcriptome sequencing of the control group. E) Violin plots showing the expression of MCAM and NR2F2 in the identified 10 cell clusters in the control group. F) Spatial heatmap showing the expression of MDK in the control sample. The red wireframe area is the area of inferred stem/progenitor cell distribution. G) HE staining of spatial transcriptome sequencing samples. H) Spot light showing subpopulations of chondrocyte and FSPC proportion in control and DDH samples.

For a deeper and multi‐angle interpretation of the molecular mechanisms driving disease progression in DDH through the acetabular labrum, we performed spatial transcriptome sequencing on control and DDH samples. Initially, HE staining revealed a disordered fiber structure in the DDH group (**Figure** **5**G). Analysis identified seven clusters in the DDH group and ten clusters in the control group (Figure 5A,D). Next, we investigated the expression level of *MCAM* and *NR2F2*, marker genes of FSPCs. Combined with the results of DEGs, cluster 4 in the DDH group and cluster 5 in the control group were identified as potential FSPCs (Figure 5B,E).

Analyzing *MDK* expression in the two spatial transcriptome sequencing samples, we found that in the DDH group, the spatial distribution of high *MDK* expression areas largely overlapped with cluster 4, while in the control group, the region with high *MDK* expression was distant from cluster 5. Furthermore, chondrocyte‐related data from scRNA‐seq were mapped onto the spatial transcriptome by deconvolution. Compared with the control group, the DDH group exhibited more complex cell types and a higher distribution of Ch8. Additionally, the distribution of FSPCs highly coincided with that of Ch8, facilitating interaction among the clusters. Thus, our scRNA‐seq results were further validated at the spatial transcriptome level.

### 2.7 Inhibition of MDK can Delay the Progression of DDH in Rats

**Figure 6:**
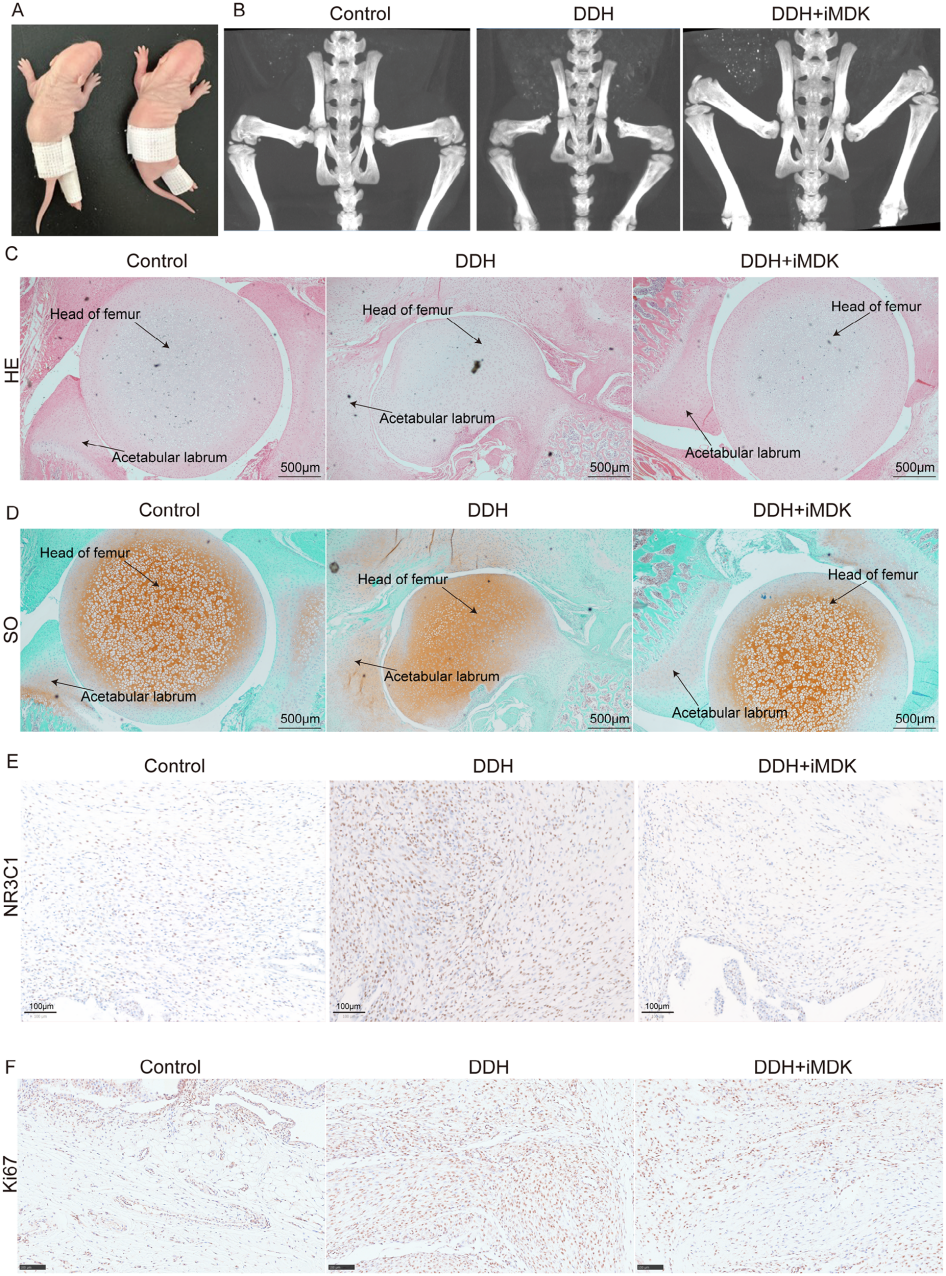
In vivo experiments were performed to verify the role of MDK in the progression of DDH. A) Establishment of the DDH model in rats. B) Micro‐CT examination of the hip joint of rats in different groups. C,D) HE and Safranin O and fast green staining of the hip joint of rats in different groups (the same joint slice plane). E,F) Immunohistochemical staining of NR3C1 and Ki67 in the hip joint of rats in different groups.

To thoroughly validate the results obtained from sequencing and in vitro experiments, we established the rat model of DDH (**Figure** **6**A). Following 24 days of various treatments, hip joint specimens were collected for µCT examination. The results indicated that compared with the control group, the acetabulum in the DDH group became shallow, and the hip joint was dislocated. However, with iMDK treatment, the dislocation of the hip joint was notably improved (Figure 6B). HE and Safranin O and fast green staining revealed acetabular labrum thickening and joint structural disorder in the DDH group, while the iMDK intervention group exhibited inhibition of these abnormal findings within the joint (Figure 6C,D). Immunohistochemistry results showed that the expression of NR3C1 and Ki67 in the DDH group was the highest, followed by the DDH+iMDK group, with the control group exhibiting the lowest expression of these three proteins (Figure 6E,F). These findings further support the notion that MDK inhibition could suppress acetabular labrum thickening and thereby inhibit or delay the progression of DDH.

## 3 Discussion

DDH poses a significant challenge to the health of infants and young children, often leading to the onset of hip osteoarthritis in adulthood. Previous researches on DDH have predominantly focused on abnormalities in the development of the hip joint osseous structures. Currently, most existing clinical treatment strategies, such as surgery, primarily target the aforementioned issues, limited to alleviating the late‐stage symptoms of DDH, without addressing underlying pathobiology problems.^[^ ^27^ ^]^ However, the significant trauma, complications, and high medical costs associated with surgery often make it difficult for patients and their families to accept. Therefore, the development of novel treatments such as molecular therapy and targeted therapy has become an urgent issue in the field of DDH treatment and holds significant clinical significance. In order to develop new treatment methods, it is necessary to delve into the underlying pathogenesis of DDH. Increasingly, researches support the pivotal role of changes in the acetabular labrum in the occurrence and development of DDH. However, the underlying mechanisms of pathological changes in the acetabular labrum in DDH remain unclear. Therefore, we collected acetabular labrum specimens and constructed the first single‐cell atlas of the human acetabular labrum through single‐cell combined spatial transcriptome sequencing analysis.

Chondrocytes are considered the primary cell type in the acetabular labrum and are central mediators of tissue degeneration and DDH. We identified eight chondrocyte subclusters through scRNA‐seq. Further subcluster analysis delineated the characteristics of each subcluster, and combined with cell proportion analysis, revealed a significant increase in ossification‐related chondrocyte subclusters in the DDH group, indicating that ossification is a critical event in the disease process. Based on these results, we confirm the severe degeneration of the acetabular labrum in the DDH group, consistent with clinical observations. This may be due to increased weight‐bearing on the acetabular labrum due to hip joint dislocation and abnormal hypertrophy of the acetabular labrum in DDH patients, resulting in greater wear and tear.^[^ ^28^ ^]^ Pseudotime analysis also revealed a chondrocyte subcluster trajectory that clearly reflects the degenerative process of the acetabular labrum.

In the field of orthopedic tissue engineering, stem/progenitor cells have become a hot topic of research.^[^ ^29^ ^]^ Stem/progenitor cells have been reported to exist in fibrocartilage tissues similar to the acetabular labrum, such as the meniscus and intervertebral disc.^[^ ^17^, ^30^ ^]^ In our study, we also discovered the presence of stem/progenitor cells in the human acetabular labrum and successfully isolated them. Currently, researches on stem/progenitor cells mainly focus on their application in tissue repair and disease treatment.^[^ ^31^ ^]^ However, in the pathogenesis of DDH, we speculate that the abnormal hypertrophy of the acetabular labrum may be associated with excessive proliferation of stem/progenitor cells. First, we conducted DEGs analysis on the two identified Stem/progenitor cell subclusters, FSPC1 and FSPC2, revealing that FSPC2 accounts for a larger proportion of DDH and is closely related to immune cell chemotaxis. For this DEGs analysis, we also compared outcomes with and without including sex as a covariate to evaluate whether sex differences could confound the results. The results illustrated that the expression of functionally relevant genes such as CXCL8, CXCL3, CCL2, and CXCL2 remained consistent regardless of sex adjustment. This can explain the increased immune cell infiltration in acetabular labial specimens in the DDH group compared to the control group. Abnormal immune cells infiltration can lead to tissue inflammation, local edema, and fibrosis, resulting in abnormal tissue enlargement. Transcription factor analysis showed high expression of *NR3C1* in FSPC2, which is closely related to cell proliferation. Studies have shown that high expression of NR3C1 can promote cell proliferation.^[^ ^32^ ^]^ In vitro experiments also demonstrated that FSPC in the DDH group has stronger proliferation capability than those in the control group. These results indicate that the enhanced proliferation of FSPC in the acetabular labrum of the DDH group is associated with the abnormal hypertrophy of the acetabular labrum and the progression of DDH.

Communication between cells can be achieved through ligand‐receptor interactions, and therefore, targeting cell–cell interactions is often used for molecular or targeted therapy. Cellchat analysis revealed that the MK signaling network in the DDH group is activated compared to the control group, with FSPCs being the main receptor cell. MDK, as the only ligand molecule in the MK pathway, is also positively correlated with cell proliferation.^[^ ^33^ ^]^ In our vitro experiments, we observed that FSPCs from the DDH group exhibited elevated expression levels of MDK and NR3C1 compared to those from the control group, along with enhanced proliferation capacity. Remarkably, this heightened proliferation tendency was effectively counteracted by the administration of iMDK, a targeted inhibitor specifically designed to inhibit MDK. Through our in vivo experiments, we further substantiated that the inhibition of MDK could effectively retard the progression of the disease in DDH rats. We acknowledge that the intra‐articular injection mode of drug delivery may result in pharmacological effects extend beyond the labrum to the surrounding joint tissues. Future studies incorporating localized drug‐tracking or tissue‐specific gene expression readouts are warranted to definitively validate site‐specific activity. Spatial transcriptome sequencing results showed that MDK molecules and FSPC cells in the DDH group are adjacent in space, which is more conducive to interaction and promotes disease progression compared to the control group, consistent with the results of scRNA‐seq. Based on these findings, we elucidated MDK as a target for early intervention, which may delay or reverse structural problems associated with DDH, thereby producing therapeutic effects on DDH. Since our intervention was conducted in juvenile rats, we believe that age may significantly influence the treatment outcome when applied to humans. If DDH is detected in infants, early intervention is crucial. Delaying treatment until adulthood, when structures like the acetabular labrum are fully developed, could greatly reduce the effectiveness of molecular therapies.

Herein, for the first time, we have employed a novel approach using single‐cell combined spatial transcriptomics to elucidate the pathogenesis of DDH from the perspective of the acetabular labrum. By integrating clinical manifestations, we identified the aberrantly activated MK signaling pathway within the acetabular labrum of DDH patients, exerting its effects on FSPC cells and leading to their abnormal proliferation. This elucidation offers a pioneer molecular‐level understanding of the pathological manifestations of the acetabular labrum in DDH patients. Through comprehensive in vivo and in vitro experiments, we have not only validated that targeted inhibition of MDK can mitigate or even reverse early structural abnormalities in the hip joint linked with DDH, thereby triggering a therapeutic response, but also holds profound implications for DDH treatment. Hence, we furnish a robust theoretical framework for the early pharmacological management of DDH and pave the way for the clinical translation of pharmacological interventions for DDH.

## 4 Conclusion

In conclusion, our groundbreaking study has spearheaded the creation of the inaugural cellular atlas of the human acetabular labrum, establishing an indispensable cornerstone for unraveling the complexities of DDH pathogenesis and unveiling transformative therapeutic strategies. By meticulously identifying pathogenic cellular subpopulations and pivotal signaling pathways in DDH, we have illuminated unprecedented and promising avenues for its treatment. Moreover, our rigorous validation of the central role of the MK signaling pathway in DDH pathogenesis through comprehensive in vivo and in vitro experiments not only underscores but amplifies the potential of localized drug interventions as an innovative and early treatment modality for DDH. Consequently, our research carries profound and far‐reaching clinical implications, laying down essential and pioneering groundwork for the evolution of molecular therapies in DDH. It introduces a groundbreaking non‐invasive treatment option, poised to revolutionize the prevailing clinical treatment landscape and set a new gold standard in care for DDH patients.

## 5 Experimental Section

### Biological Samples

Ten patients with osteonecrosis of the femoral head and ten patients with DDH who were admitted to our hospital and met the inclusion criteria were included. The general clinical and demographic data of the osteonecrosis of the femoral head patient group and DDH group are shown in Table S1 (Supporting Information). All patients were hospitalized. Acute episodes of osteonecrosis of the femoral head or DDH lasted no less than 3 days, and the duration of the disease was more than 1 year. Of the 10 patients with osteonecrosis of the femoral head (mean age value = 55.6), 7 are male and 3 are female. Of the 10 patients with DDH (mean age value = 51.8), 3 are male and 7 are female. The difference of the mean vales of BMI, ESR, CRP, ALT, AST, and Urea between the two groups was not statistically significant. The mean IL‐6 (7.187 vs 3.571; p = 0.0321) and Creatinine (72.9 vs 58.3; p = 0.0301) in the osteonecrosis of the femoral head group were higher than those in the DDH group.

### Human Acetabular Labrum Cell Sample Preparation

The specimens of acetabular labrum were collected from four patients with osteonecrosis of the femoral head (the control group) and four patients with DDH (the DDH group) (Table S2, Supporting Information). The experiments were approved by the Ethics Committee of Sichuan University (Ethics Committee on Biomedical Research, West China Hospital of Sichuan University No. 2020‐(921)). The following is a description of our consent procedures. We submitted ethical documents to the Ethics Committee at West China Hospital of Sichuan University for review, attended their meeting for evaluation, and obtained approval upon successful assessment.

All specimens removed were placed immediately in normal saline free of antibiotics under 4 °C. The specimens were rinsed in 1 × PBS and then were cut into 1 mm^3^ pieces. 3 mg mL^−1^ Collagenase P (Roche 11213873001) was used to digest the tissues under 37 °C for 3–4 h. Next, Ham's F‐12 media containing 10% FBS was applied to stop the process of digestion, and then a 100 µm cell strainer was used to filter the digested tissue. Lastly, after centrifugation, cells were harvested for subsequent batch analysis.

### Processing of scRNA‐Seq Data

Using CellRanger count version 4.0.0, the sequencing data were aligned to the human reference genome GRCh38 to obtain a single‐cell level gene expression matrix. Subsequently, the gene expression matrix was imported into a Seurat object for further downstream analysis (version 4.3.0). To ensure the quality of the cells used in subsequent analyses, a rigorous quality control (QC) pipeline is implemented. Specifically, for each sample, DoubletFinder was used to predict droplets that might encapsulate multiple cells, and only cells predicted as “Singlet” were retained. Cells exhibiting high proportions of mitochondrial gene expression (>20%) are filtered out as well as a high percentage of transcripts that map to dissociation‐induced genes (>10%), which refer to the previous study.^[^ ^34^ ^]^ Additionally, cells with low Unique Molecular Identifiers (UMI) counts (<500), gene counts (<200), and a log10GenesPerUMI value of less than 0.8 are also excluded.

See Supporting Information Appendix, Supplementary Methods (Supporting Information) for detailed descriptions of data analyses and additional assays performed in this study.

## Conflict of Interest

The authors declare no conflict of interest.

## Author Contributions

R.Y., H.L., and M.G. contributed equally to this work. W.F., X.M., and J.L. conceived the project. W.F. and X.M. designed the experiments. R.Y., M.G., T.X., L.Z., and Y.Z. collected the specimens. H.L., R.Y., M.G., and D.Z. performed data collection and analysis. R.Y., X.M., and S.L. performed in vitro and in vivo experiments. X.M., W.F., and R.Y. wrote the manuscripts. All authors commented and revised the manuscripts.

## Supporting information



Supporting Information

## Data Availability

The data that support the findings of this study are available from the corresponding author upon reasonable request.
