# Supplementary material for: Deciphering the Acetabular Labrum's Cellular Atlas: MDK Inhibition as a Novel Therapeutic Method for Developmental Dysplasia of the Hip
Source: Adv Sci (Weinh). 2025 Jul 2;12(37):e05803. doi: 10.1002/advs.202505803 (PMC12499403; doi:10.1002/advs.202505803)
Supplement: Supplementary file 1 — Supporting Information [file ADVS-12-e05803-s001.docx]

**Deciphering the Acetabular Labrum's Cellular Atlas: MDK Inhibition as a Novel Therapeutic Method for Developmental Dysplasia of the Hip**

Runze Yang^1#^, Huiling Liu^3#^, Minghao Ge^1#^, Dingyuan Zhang^3^, Tianhao Xu^1^, Lei Zhang^1^, Yanlin Zhu^1^, Shun Li^2^, Jian Li^1^, Xin Ma^2,3,4*^, Weili Fu^1*^

^1^ Sports Medicine Center, Department of Orthopedic Surgery/Orthopedic Research Institute, West China Hospital, Sichuan University, 610064 Chengdu, Sichuan, China

^2^ School of Life Science and Technology, University of Electronic Science and Technology of China, 610054 Chengdu, China

^3^ Tianfu Jiangxi Laboratory, 641419 Chengdu, Sichuan, China

^4^ GenomCan Inc., Chengdu, Sichuan, China

#These authors contributed equally to this work.

*Correspondence: [foxwin2008@163.com](mailto:foxwin2008@163.com) (W. F.); [xm24@uestc.edu.cn](mailto:xm24@uestc.edu.cn) (X. M.)

Supplementary Methods:

Flow cytometry and cell sorting

In order to sort out the FSPCs, firstly, the collected acetabular labrum tissue was carefully cleaned to remove blood vessels, synovium, and other mixed tissues. Secondly, cells digested from the acetabular labrum were incubated with CD146 (361012, Biolegend) for 60 minutes. After that, BD FACS Aria III was used to sort CD146^+^ cells.

Colony-forming and multi-differentiation potential assays

The sorted cells were seeded at a low density to form colonies on a 6-well plate (1000 cells/well). 7 days later, 0.1% crystal violet was used to stain the colonies.

For multi-differentiation assays, sorted cells at 70% to 80% confluence in 6‐well plates were treated with osteogenic differentiation induction medium (HUXMA90021, Cyagen Biosciences) for 7 days, or adipogenic differentiation induction medium (HUXMA-90031, Cyagen Biosciences) for 21 days. Alizarin Red staining was performed for osteogenic differentiation and Oil Red staining was performed for adipogenic differentiation.

Immunohistochemistry

Immunohistochemical staining was performed as described previously^1^. Tissue slices were deparaffinized and rehydrated. Next, for antigen retrieval, slices in 0.1% EDTA solution were treated with a microwave for 10 minutes. After that, 3% hydrogen peroxide was applied to block the endogenous peroxidase activity and 5% BSA was used to block the nonspecific binding sites under 37℃. The primary antibodies (CD146 (A13927, ABclonal), MDK (A0251, ABclonal), NR3C1 (A2164, ABclonal), Ki67 (A20018, ABclonal)) were incubated with sections overnight at 4℃. The next day, sections were incubated with the appropriate HRP-conjugated secondary antibody and counterstained with hematoxylin. Semi-quantitative analysis of the immunohistochemistry results was performed by using image J software.

Western blot

We used the same method as before to perform Western blotting^2^. Briefly, cells were seeded onto the 6-well plate and stimulated with 0.5 Μm Imdk (HY-110171, MedChemExpress) for 72h. After that, the cells were collected and washed with sterile PBS, followed by the treatment with 100 μL of radioimmunoprecipitation (RIPA; Beyotime) assay buffer containing phenylmethylsulfonyl fluoride (PMSF; Beyotime) to harvest the proteins in cells. After calculating the protein concentration by using a bicinchoninic acid (BCA) assay kit (Beyotime), 30 μg of proteins were resolved in 12.5% sodium dodecyl sulphate-polyacrylamide gel electrophoresis (SDS-PAGE) and transferred to the PVDF membranes (Millipore, USA). Then the membrane was treated with 5% skim milk to block the nonspecific proteins at room temperature for 60 minutes, incubated with specific primary (MDK (A0251, ABclonal), NR3C1 (A2164, ABclonal)) antibodies at 4°C overnight, and stained with the secondary antibodies for 1 h. Protein bands on the membranes were visualized using the ChemiDocTM XRS+ System (Bio-Rad Laboratories, USA) with a chemiluminescence substrate kit (Oriscience Biotechnology, China).

EdU cell proliferation assay

Cell proliferation was assessed using the EdU cell proliferation assay in accordance with the manufacturer's guidelines. Approximately 1 × 10^5^ cells were seeded into 12-well plates and incubated for 24 hours before the assay. Each well was then treated with 500 µL of EdU (10 µM) reagent (C0071S, Beyotime) and incubated for 2 hours to label the cells. After three washes with PBS, the cells were fixed in a 4% paraformaldehyde solution for 20 minutes, permeabilized with 0.3% Triton X-100 for an additional 10 minutes, and then incubated with the click-reaction reagent for 30 minutes at 37℃ in the dark. Finally, the nuclei were counterstained with 1× Hoechst33342 reagent. The staining results were observed using a Nikon ECLIPSE Ti-S fluorescence microscope system.

Animals and establishment of the DDH model

All animal experiments were performed following the Guide for the Care and Use of Laboratory Animals published by the Chinese National Academy of Sciences and were approved by the Animal Ethics Committee of West China Hospital, Sichuan University. A total of 15 newborn Wistar rats were divided into three groups (Control, DDH, iMDK). DDH was induced in newborn rats following established protocols as described previously^3,4^. In brief, DDH was induced by immobilizing the hind legs of rats in the DDH and iMDK groups using medical tape and positioning them in hip adduction and extension for 10 days. No intervention was performed on newborn rats in the normal control group. All newborn rats were nourished by their mothers and showed typical growth and nutritional status. Rats in the DDH and iMDK groups were released from the medical tape for 30 minutes each day. After 10 days, rats in the iMDK group were treated with 5 mg/kg iMDK (HY-110171, MedChemExpress) in the hip joint for 7 days, and the DDH and control groups were injected with an equal volume of saline. Our intra-articular injection protocol was performed under anesthesia, with the injection site located midway between the greater trochanter and iliac crest. Using a 1 mL insulin syringe, the needle was introduced at a 45° ventral angle to a depth of approximately 3 mm. After confirming no blood aspiration, the agent was administered slowly; the needle was retained in place for 10 seconds to prevent reflux, followed by rapid withdrawal and 1-minute compression for hemostasis.

Microcomputed tomography (micro-CT) assay

One week after the intervention, the rats were euthanized using an intraperitoneal overdose injection of pentobarbital, followed by fixation of the samples in a 4% phosphate-buffered formalin solution. Furthermore, each group's samples underwent examination using a microcomputed tomography (micro-CT) system (μCT-100, Scanco, Switzerland), following the methodology outlined in previous studies^5,6^.

scRNA-seq Data Analysis

To construct a comprehensive single-cell atlas, all samples were merged, including those from the semilunar cartilage normal donor. The SCTransform normalization was applied, and then the top 3000 variable genes excluding mitochondrial, ribosomal, and hemoglobin genes were used for PCA dimension reduction. Next, Run Harmony with paras group.by.vars = "sample", assay.use = "SCT" to remove batch effects. The top 16 PCs were chosen for FindNeighbors to compute KNN nearest distances, followed by FindClusters for unsupervised clustering. Non-linear dimensionality reduction t-SNE or UMAP was performed for single-cell data visualization, consistent with the unsupervised clustering, with a resolution of 0.5 chosen for both clustering and non-linear dimension reduction. To identify specific cell types, the FindAllMarkers function was applied to pinpoint marker genes for clusters derived from unsupervised clustering. SingleR was used for automatic annotation based on the built-in HumanPrimaryCellAtlasData dataset to assist in completing the annotation of cell major classes.

To delve deeper into the degeneration process of chondrocytes and the regulatory mechanisms of FSPCs in DDH, specific cell subsets are extracted for a more in-depth analysis. This involves repeating the SCTransform normalization, dimension reduction, batch effect removal, clustering, and marker gene identification steps. For this subset analysis, a resolution of 0.5 is maintained for clustering and non-linear dimensionality reduction, using the top 14 harmony PCs for calculating cell distances.

Differential Gene Expression Analysis

Differential gene expression analyses were conducted to compare control individuals with DDH patients and between cell type subcluster markers using the FindAllMarkers or FindMarkers functions within Seurat. The default criteria included log-transformed fold change values greater than 0.25, adjusted P values less than 0.05, and gene expression in more than 10% of cells. These analyses were performed using MAST, which employs a specialized hurdle model tailored for scRNA-seq data unless specified otherwise.

Trajectory Analysis

For trajectory analysis, we employed the R package Monocle3 (version 1.3.1). UMAP embeddings and cell clusters, previously generated via Seurat, were converted into a cell_data_set object using SeuratWrappers, which can be smoothly incorporated into Monocle3. The analysis targeted both normal and degenerated chondrocytes from DDH patients, along with those from healthy donors. Following the re-clustering of the cells with Monocle3, trajectory analysis was executed using the learn_graph function. Genes showing variations along the trajectory were pinpointed using the graph_test function parameter neighbor_graph="principal_graph", which evaluates the correlation of gene expression among cells located similarly along the trajectory.

Velocyto was employed to obtain the count matrices of pre-mature (unspliced) and mature (spliced) transcript abundances from the raw aligned bam generated by cellranger count. Then the analysis of RNA velocity was performed by scVelo, fallowing the user guide, with default parameters. To get comparable view with previous trajectory result from monecle3, the result was embedded to consistent UMAP coordinates.

Transcription Factor Regulon Analysis

To predict the potential transcriptional regulatory network in FSPCs, we conducted SCENIC analysis using pySCENIC. The input matrix for FSPCs was the normalized expression matrix obtained from Seurat. Regulon activity, measured in AUC, was assessed using the AUCell module of pySCENIC with default thresholds. Differential expression of regulons was identified using the Wilcoxon rank-sum test within the FindAllMarkers function in Seurat.

Cell-cell Interaction Analysis

Cell-cell interactions based on the expression of known ligand-receptor pairs across different cell types were inferred using CellChat (v1.6.1). We followed the official tutorial to identify potential cell-cell communication networks between FSPCs and chondrocytes in DDH degeneration. Specifically, we loaded the normalized counts of the associated cell groups into CellChat and applied preprocessing functions, including identifyOverExpressedGenes, identifyOverExpressedInteractions, and projectData, with standard parameter settings. For the primary analyses, the core functions computeCommunProb were employed with the parameter set population.size=TRUE, operating under the assumption that abundant cell populations tend to collectively transmit stronger signals than rare cell populations. Additionally, computeCommunProbPathway and aggregateNet were applied with standard parameters. Lastly, to identify significant signaling changes between DDH and normal degeneration, we utilized multiple comparison and visualization functions in CellChat, such as compareInteractions, netVisual_heatmap, and rankSimilarity.

Function Analysis

We performed gene set enrichment analyses, including enrichGO, GSEA, and enrichPathway in Reactome using the clusterProfiler package (version 3.18.1).

Processing of Spatial Transcriptomics Data

For spatial transcriptomics of acetabular labrum samples, the tissue is first fresh-frozen and sectioned into 5μm slices using a cryostat. The sections are then placed on spatially barcoded slides for RNA capture, followed by cDNA synthesis and library preparation for sequencing. The raw sequencing reads from spatial transcriptomics underwent quality checks and was mapped to the pre-built human reference genome GRCh38-2020-A-2.0.0 using Space Ranger v2.0.1 with default settings. The resulting gene-spot matrices from Space Ranger were imported into a Seurat object for further quality control and analysis using the Seurat package (version 4.3.0). We filtered spots based on a minimum detection of 200 genes and a minimum UMI count of 500, while removing genes expressed in fewer than 10 spots. Additionally, spots with more than 10% mitochondrial counts or 20% ribosomal counts were excluded.

Spatial Transcriptomics Data Analysis

We applied SCTransform normalization across the filtered spots and conducted clustering at a resolution of 0.8, utilizing the first 30 principal components (PCs) from principal component analysis (PCA). Cells were visualized using the uniform manifold approximation and projection (UMAP) algorithm at the same dimensions used in clustering. Spatial feature expression plots were generated using the SpatialFeaturePlot and VlnPlot functions in Seurat (version 4.3.0).

To assign cell types to the labrum samples in Spatial Transcriptomics, we employed robust cell type decomposition (RCTD) in full mode. This method allows for the assignment of multiple cell types per spot and is particularly recommended for platforms like the 10x Genomics Visium. The vizAllTopics function in STdeconvolve (version 1.6.0) was utilized to visualize the proportionate weight of each cell type within individual spots.

Statistical analysis

Analysis of the data was performed with the statistical programming language R (4.0.3) using package specified in the respective sections in Methods or GraphPad Prism (version 5.0). Statistical significance was defined as follows: *p < 0.05, **p < 0.01, ***p < 0.001. To generate plots, packages ggplot2(v3.5.0), ComplexHeatmap(v2.18.0), scCustomize(v2.1.2), cowplot(v1.1.3) with R(version 4.3.3) were used in this work.

1. Yang RZ, Xu WN, Zheng HL, et al. Involvement of oxidative stress-induced annulus fibrosus cell and nucleus pulposus cell ferroptosis in intervertebral disc degeneration pathogenesis. *J Cell Physiol* 2021; **236**(4): 2725-39.

2. Yang X, Chen Y, Guo J, et al. Polydopamine Nanoparticles Targeting Ferroptosis Mitigate Intervertebral Disc Degeneration Via Reactive Oxygen Species Depletion, Iron Ions Chelation, and GPX4 Ubiquitination Suppression. *Adv Sci (Weinh)* 2023; **10**(13): e2207216.

3. Wang E, Liu T, Li J, et al. Does swaddling influence developmental dysplasia of the hip?: An experimental study of the traditional straight-leg swaddling model in neonatal rats. *J Bone Joint Surg Am* 2012; **94**(12): 1071-7.

4. Liu J, Bao Y, Fan J, Chen W, Shu Q. Microstructure changes and miRNA-mRNA network in a developmental dysplasia of the hip rat model. *iScience* 2024; **27**(4): 109449.

5. Bouxsein ML, Boyd SK, Christiansen BA, Guldberg RE, Jepsen KJ, Müller R. Guidelines for assessment of bone microstructure in rodents using micro-computed tomography. *J Bone Miner Res* 2010; **25**(7): 1468-86.

6. Wang X, Wu Q, Zhang R, et al. Stage-specific and location-specific cartilage calcification in osteoarthritis development. *Ann Rheum Dis* 2023; **82**(3): 393-402.


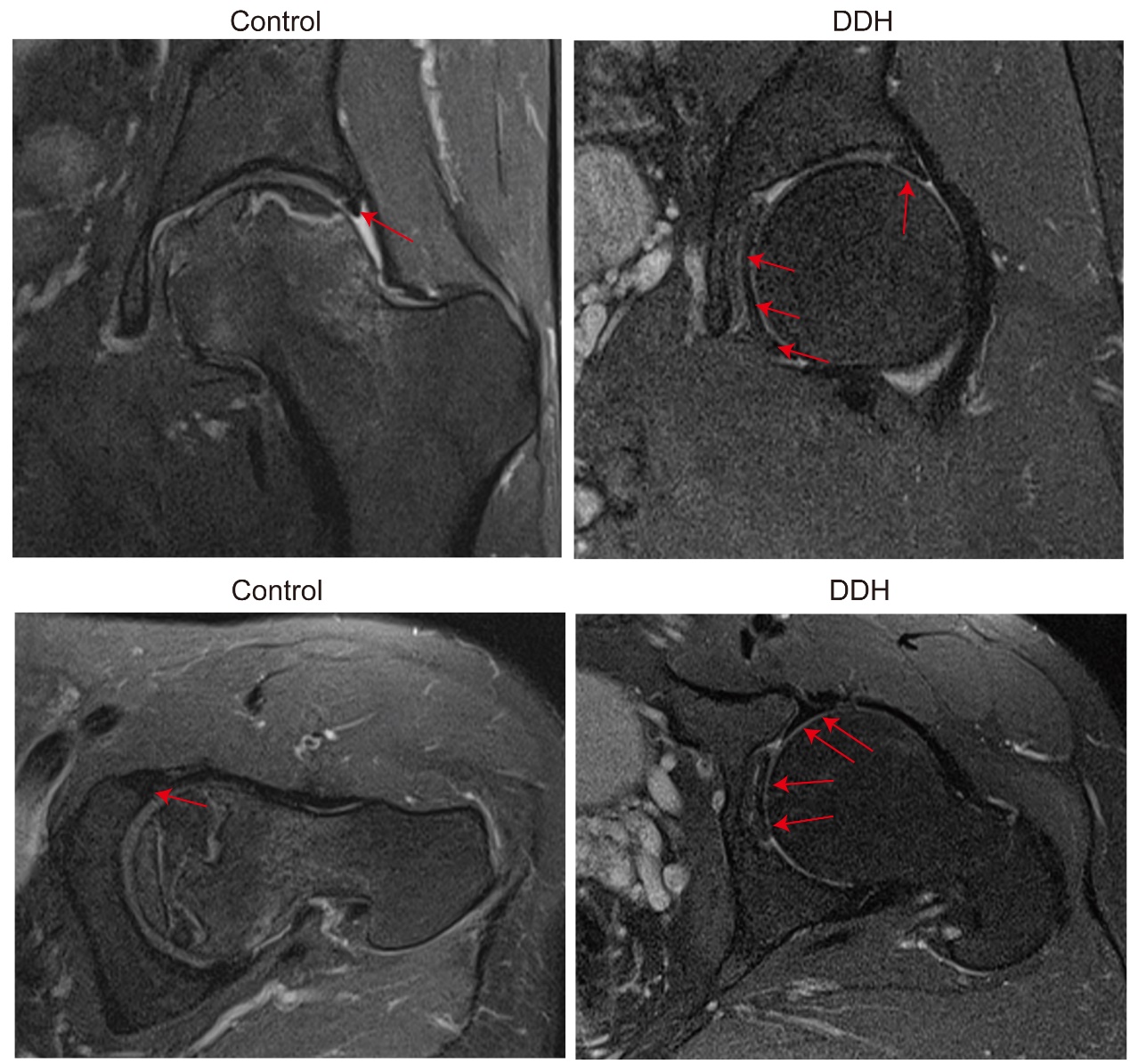


Supplementary Figure 1: MRI Photographs of typical control and DDH hip joints. The red arrow points to the acetabular labrum.


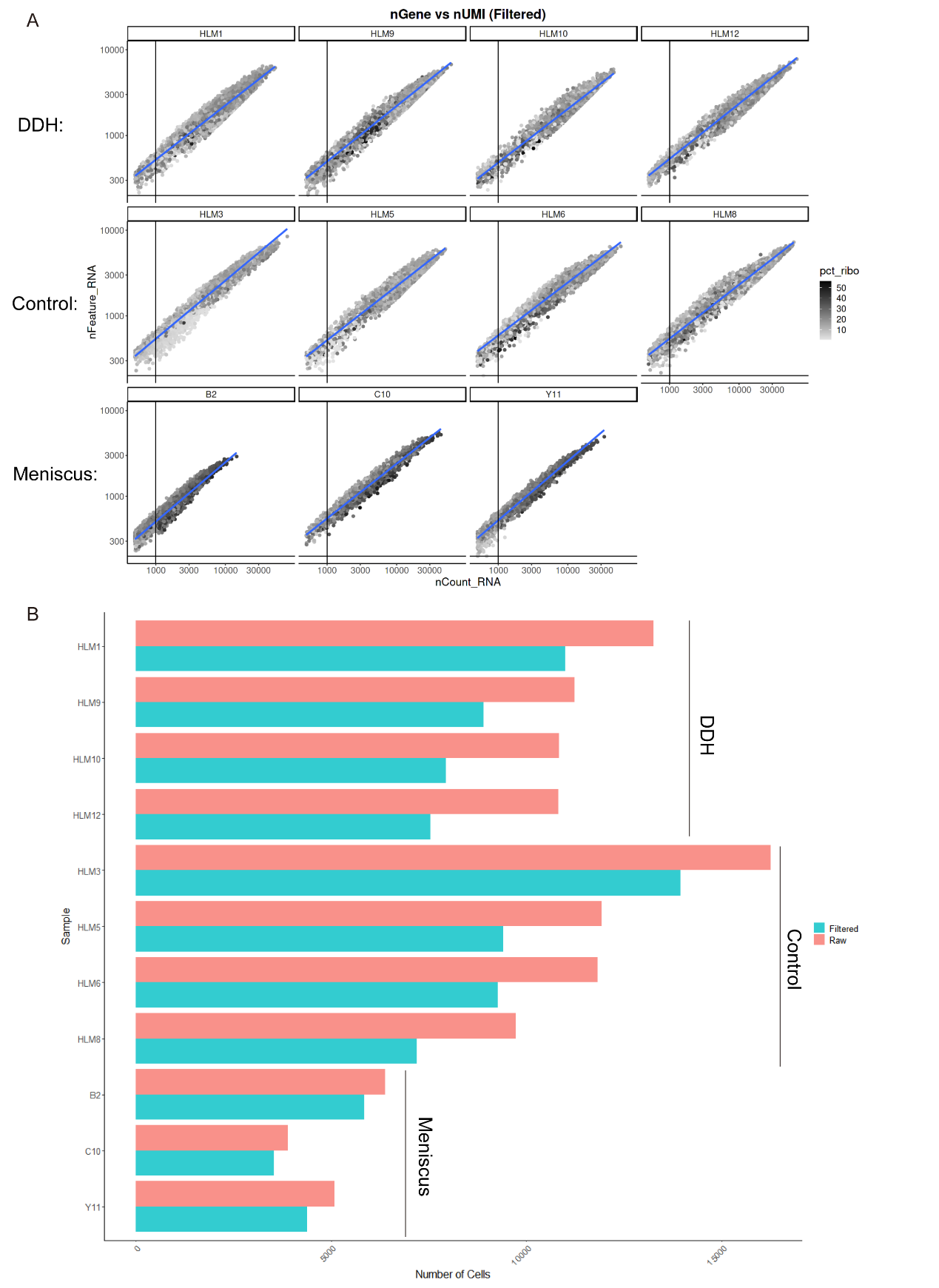


Supplementary Figure 2: A: the correlation between genes detected (nFeature_RNA) and number of UMIs (nCount_RNA) B: The number of cells in each sample before and after filtration.


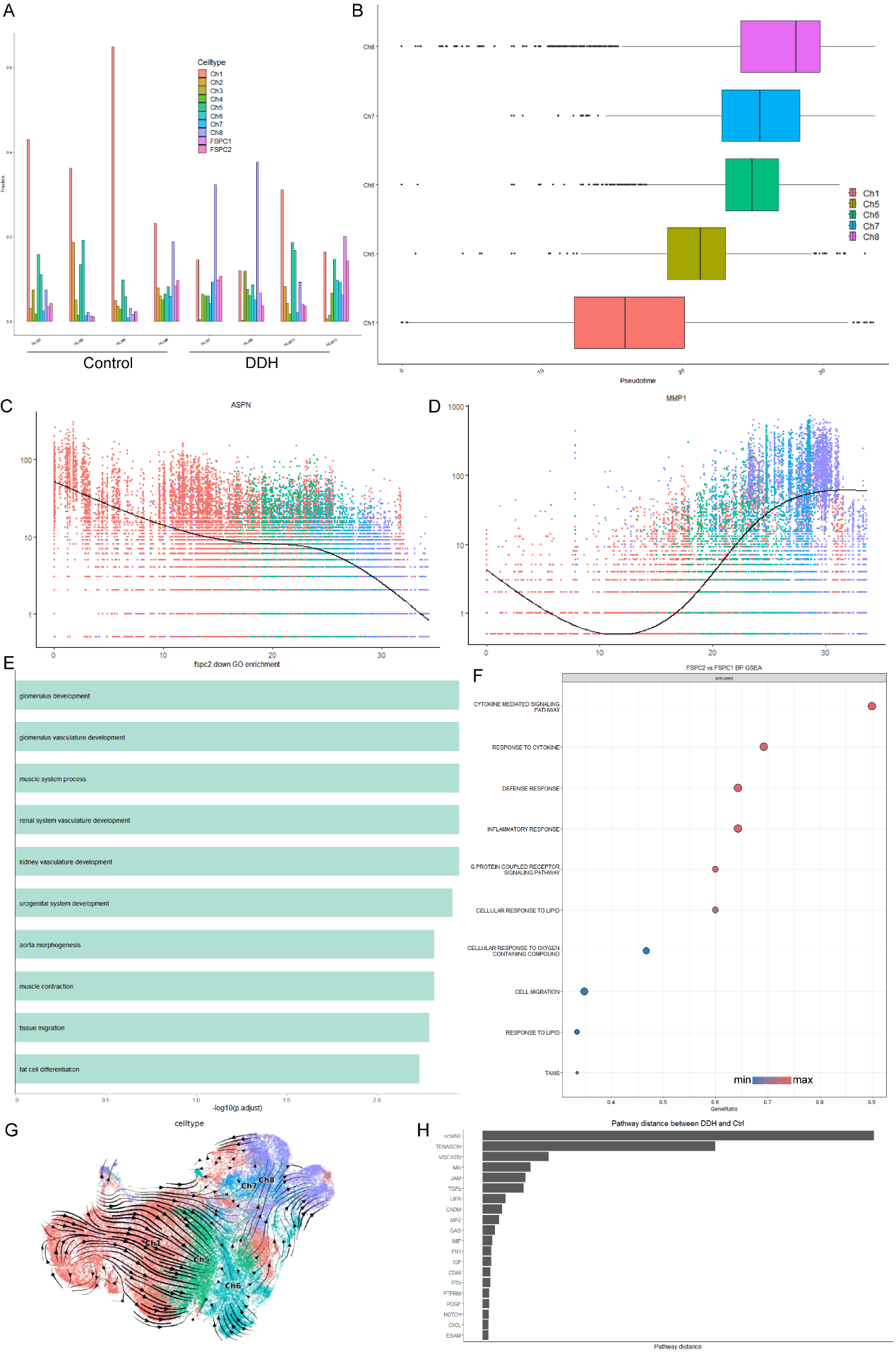


Supplementary Figure 3: A: The proportion of chondrocyte and FSPC subclusters in each sample of control and DDH groups. B: Distribution of Ch1, Ch5, Ch6, Ch7, and Ch8 in pseudotime analysis. C and D: Representative gene expression levels along the trajectory of the degeneration of acetabular labrum. E: GO enrichment analysis of Degs highly expressed by FSPC1. F: GSEA enrichment analysis of Degs between FSPC2 and FSPC1. G: RNA velocity analysis of Ch1, 5, 6, 7, and 8 with velocity field projected onto the UMAP plot. Arrows show the local average velocity evaluated on a regular grid and indicate the extrapolated future states of cells. H: The difference in interaction pathways between the DDH group and the control group.


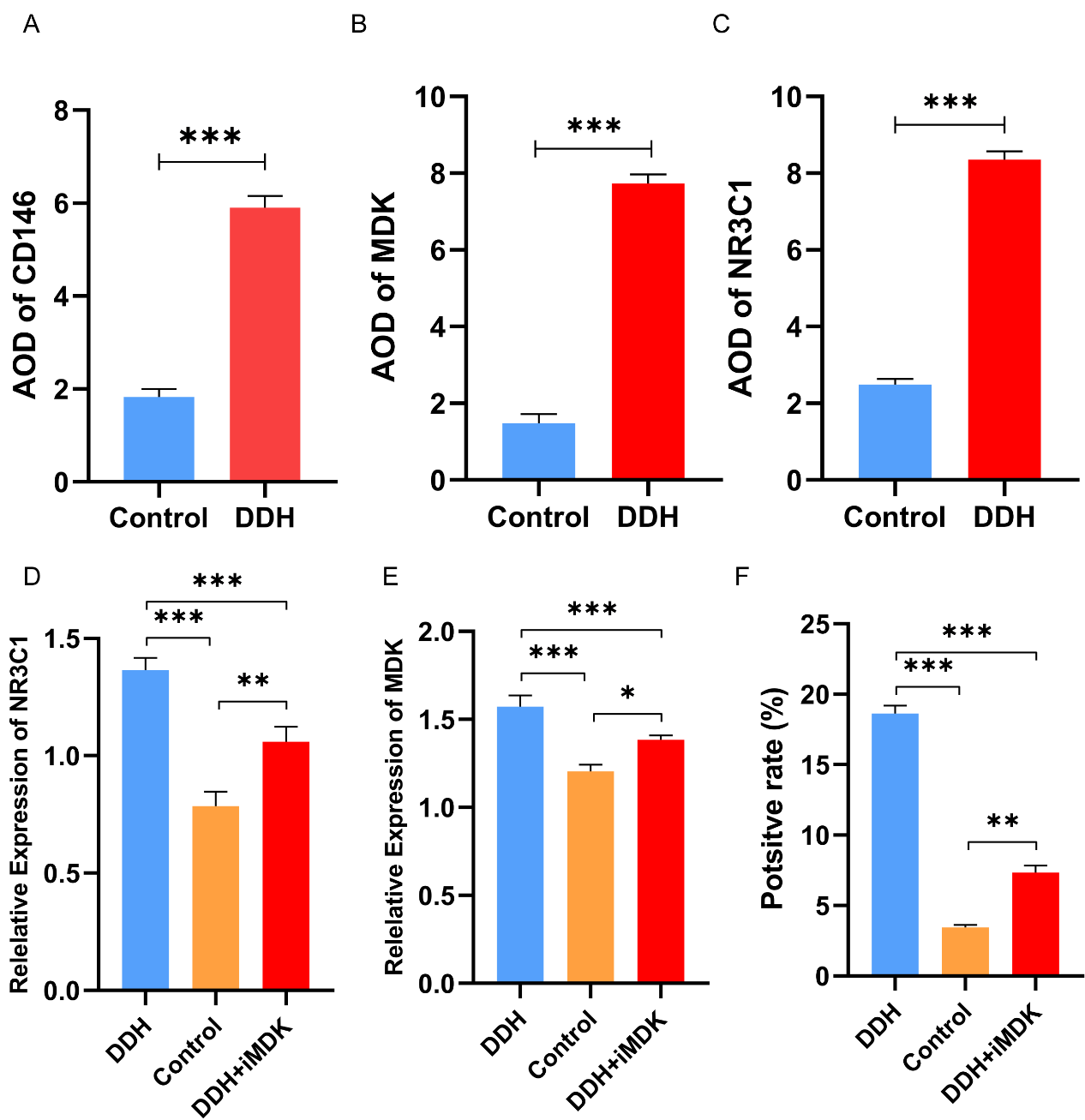


Supplementary Figure 4: A-C: Quantitative analysis of CD146, MDK and NR3C1 immunohistochemistry. D and E: Quantitative analysis of western blot of NR3C1 and MDK. F: Statistical graph of the EdU assay. N=3. *p < 0.05, **p < 0.01, ***p < 0.001.

Supplementary Table1: Population and clinical characteristics of patients

|  | Osteonecrosis of the Femoral Head | Developmental dysplasia of the hip | P value |
| --- | --- | --- | --- |
| Number | 10 | 10 |  |
| Gender | Male: 7; Female: 3 | Male: 3; Female:7 |  |
| Age(years) | 55.6 | 51.8 | 0.5671 |
| BMI(kg/m^2^) | 24.25 | 23.75 | 0.751 |
| ESR(mm/h) | 42.6 | 41.2 | 0.9044 |
| CRP(mg/l) | 7.201 | 8.019 | 0.8639 |
| IL-6(pg/ml ) | 7.187 | 3.572 | 0.0321 |
| ALT(U/L) | 23.3 | 22.3 | 0.8872 |
| AST(U/L) | 26 | 21 | 0.1937 |
| Creatinine(μmol/L) | 72.9 | 58.3 | 0.0301 |
| Urea(mmol/L) | 5.78 | 5.18 | 0.5928 |

Supplementary Table2: Donor and sample information

| sample name | donor age | donor sex | donor status | Sequencing type |
| --- | --- | --- | --- | --- |
| HLM1 | 69 | male | DDH | scRNA-seq and spRNA-seq |
| HLM9 | 57 | female | DDH | scRNA-seq |
| HLM10 | 78 | female | DDH | scRNA-seq |
| HLM12 | 63 | female | DDH | scRNA-seq |
| HLM3 | 52 | male | osteonecrosis of the femoral head | scRNA-seq |
| HLM5 | 68 | male | osteonecrosis of the femoral head | scRNA-seq |
| HLM6 | 69 | female | osteonecrosis of the femoral head | scRNA-seq |
| HLM8 | 53 | male | osteonecrosis of the femoral head | scRNA-seq and spRNA-seq |
